# AI-integrated smartwatch monitoring for early detection of stroke and hemorrhage: A systematic review

**DOI:** 10.1097/MD.0000000000047775

**Published:** 2026-02-20

**Authors:** Muhammad Mohsin Khan, Noman Shah, Javed Iqbal, Brijesh Sathian, Simbarashe Samakande, Bipin Chaurasia

**Affiliations:** a Department of Neurosurgery, Hamad Medical Corporation, Doha, Qatar; b Nursing Department, Hamad Medical Corporation, Doha, Qatar; c Geriatrics and Long-Term Care Department, Hamad Medical Corporation, Doha, Qatar; d Department of Neurosurgery, Parirenyatwa Group of Hospital, Harare, Zimbabwe; e Department of Neurosurgery, College of Medical Sciences, Bharatpur, Nepal.

**Keywords:** artificial intelligence, intracranial hemorrhage, machine learning, smartwatch, stroke detection, subarachnoid hemorrhage, wearable, wearable sensors

## Abstract

**Background:**

Early detection of cerebrovascular events (subarachnoid hemorrhage [SAH], ischemic stroke, intracranial hemorrhage [ICH]) is critical for timely intervention. Emerging smartwatches with multiple sensors and artificial intelligence (AI) algorithms offer potential for real-time detection of acute neurological events. We performed a systematic review (2010–2025) per the Preferred Reporting Items for Systematic Reviews and Meta-Analyses 2020 guidelines to identify published studies on AI-enabled smartwatches for detecting SAH, ischemic stroke, and ICH.

**Methods:**

We searched multiple databases from January 2010 through May 2025 using combinations of *smartwatch OR wearable*, *stroke OR SAH OR ICH*, and *AI OR machine learning OR deep learning*. Risk of bias was assessed using the Quality Assessment of Diagnostic Accuracy Studies-2 tool for diagnostic accuracy studies.

**Results:**

The search yielded 3 eligible studies on ischemic stroke detection by wearable accelerometers with AI. The stroke studies (n = 3) used bilateral wrist/arm accelerometers to detect unilateral motor deficits. Deep learning models achieved high diagnostic accuracy (area under the receiver operating characteristic curve 0.95–0.99) for detecting acute stroke symptoms. One study reported a median detection time of 15 to 29 minutes after stroke onset, depending on the false alarm threshold. A feasibility trial (STROKE ALARM) using accelerometer bands and a smartphone app triggered frequent false alarms without observed strokes.

**Conclusion:**

Wearable technology with AI shows promise for ischemic stroke symptom detection, but there is a critical gap in SAH and ICH detection. Challenges include sensor accuracy, false alarms, and algorithm generalizability. We propose a conceptual multisensory model integrating heart rate (electrocardiogram/photoplethysmography), blood pressure surrogates, and motion data into an AI pipeline for future smartwatch systems, which can lead to the detection of stroke/SAH, might show promise in other brain pathologies.

**Key Points**

Wearable technology combined with machine learning can detect stroke-related motor deficits with high accuracy in controlled settings. However, no analogous studies exist for subarachnoid or intracerebral hemorrhage. Real-world implementation faces hurdles: high false-alarm rates, variable sensor accuracy, and the need for multimodal data integration. Conceptually, a next-generation smartwatch system might continuously monitor heart rhythm, blood pressure surrogates, and limb movement using AI to flag emergent stroke or hemorrhage patterns.

## 1. Introduction

Stroke (ischemic and hemorrhagic) is a leading cause of mortality and disability worldwide. Early recognition of stroke symptoms and rapid hospital presentation are crucial for therapies such as thrombolysis or thrombectomy. Similarly, subarachnoid and other intracranial hemorrhages (ICH) require urgent diagnosis. Conventional detection relies on patient report or observer recognition of neurological deficits, often leading to delays. In recent years, consumer wearables (smartwatches and fitness bands) have evolved to include advanced sensors – electrocardiograms (ECG), photoplethysmography (PPG), accelerometers, gyroscopes, blood oxygen, and pressure monitors – enabling continuous physiological monitoring. Concurrent advances in artificial intelligence (AI)/machine learning (ML) allow real-time analysis of complex multivariate streams.^[1–5]^

Mobile health technologies, therefore, have the potential to “transform stroke detection” by continuously monitoring language, motor, gait, and sensory parameters indicative of stroke onset. For example, smartwatches detect atrial fibrillation (AF) to help prevent stroke; analogous AI algorithms could hypothetically recognize acute stroke signs (e.g., unilateral limb weakness detected by asymmetric motion) or precursors (e.g., hypertensive spike via PPG). A recent review highlighted high sensitivity/specificity of wearable AI systems for stroke signs but noted device accuracy variability and algorithm opacity as challenges.^[6–9]^

Despite these possibilities, the real-world evidence on smartwatch-based stroke/hemorrhage detection remains unclear. To our knowledge, no systematic review has examined AI-enhanced smartwatch monitoring for acute subarachnoid hemorrhage (SAH), ischemic stroke, or ICH detection. We therefore performed a systematic review following the Preferred Reporting Items for Systematic Reviews and Meta-Analyses (PRISMA) 2020 standards to identify and synthesize published studies (2010–2025) on this topic. We focused on commercial and research-grade smartwatches/wearables, requiring an AI/ML component, and excluded unpublished reports. Our goals were to catalog existing systems and their performance, assess study quality and bias, identify gaps and limitations, and propose future directions, including a conceptual multisensory AI-smartwatch model.

## 2. Methods

**Figure 1.**
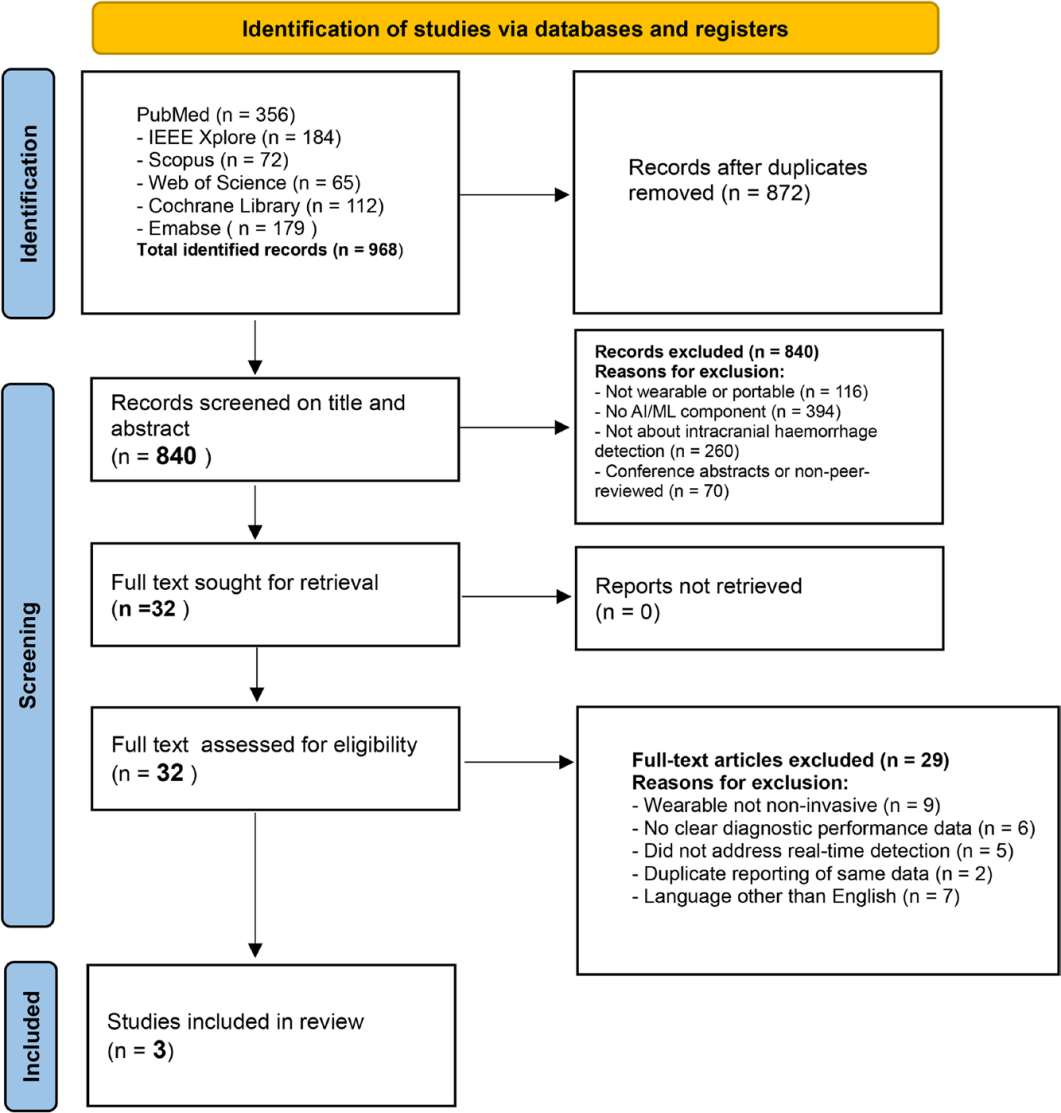
PRISMA flowchart. PRISMA = Preferred Reporting Items for Systematic Reviews and Meta-Analyses.

This review was conducted according to PRISMA 2020 guidelines. The protocol was not preregistered but adhered to standard systematic review methodology. We searched databases and followed dual-review selection with data extraction; disagreements were resolved by consensus. A PRISMA flow diagram (Fig. 1) illustrates the search and selection process.

### 2.1. Search strategy

We systematically searched PubMed/MEDLINE, Institute of Electrical and Electronics Engineers (IEEE) Xplore, ACM Digital Library, Web of Science, and Scopus from January 1, 2010, through May 15, 2025. Search terms combined device and condition keywords (e.g., *“smartwatch” OR “wearable device” OR “fitness band”*) AND *“stroke” OR “ischemic stroke” OR “brain haemorrhage” OR “intracranial hemorrhage” OR “subarachnoid haemorrhage”* AND *“artificial intelligence” OR “machine learning” OR “deep learning.” Boolean combinations and synonyms (e.g., “brain bleed”*) were used. We also hand-searched the references of relevant papers. Search filters were applied to include only peer-reviewed publications. The search strategy is summarized in Table 1.

**Table 1:** Wearable AI studies for ischemic stroke detection.

Study (yr)	Device (sensors)	Population (stroke vs controls)	AI model/algorithm	Performance (metrics)
Wasselius et al (2021)^[2]^	Bilateral wrist accelerometer bracelets (research)	84 stroke (acute hemiparesis) vs 101 healthy controls	Deep learning (FCN, inception time) and classical ML (SVM, RF)	AUC ≈ 0.95–0.99 for detecting arm paresis (15–120 min windows)
Messé et al (2023)^[4]^	Bilateral arm accelerometers (ActiGraph-type device)	33 stroke patients vs 167 controls (hospitalized)	Custom stroke detection algorithm (feature-based classifier)	Median stroke detection 15.0 min (false alarms 3.6/d) or 29.0 min (1.1/d)
Esbjörnsson et al (2022)^[3]^ (STROKE ALARM)	Wrist accelerometers + smartphone app	30 patients with stroke/TIA	Predefined motion-threshold algorithm	Not applicable (feasibility): high false-alarm rate; no strokes captured
Jeon et al (2023)^[7]^, IEEE access	IMU wristbands recorded overnight motion	46 healthy subjects and 43 stroke patients with known hemiparesis	Deep ensemble (“EDIS”) combined multiple CNNs	Sensitivity ~90.7% and specificity ~78.7%

AI = artificial intelligence, AUC = area under the receiver operating characteristic curve, CNN = convolutional neural network, EDIS = ensemble deep learning in-sleep stroke detection model, FCN = fully convolutional network, IEEE = Institute of Electrical and Electronics Engineers, IMU = inertial measurement unit, ML= machine learning, RF = random forest, SVM = support vector machine, TIA = transient ischemic attack.

- **Databases:** PubMed/MEDLINE, IEEE Xplore, Embase, Web of Science, Scopus, Cochrane Library.
- **Timeframe:** January 1, 2010 to May 15, 2025.
- **Languages:** English.
- **Keywords:** As above; examples: “smartwatch stroke detection,” “wearable accelerometer stroke machine learning,” “AI wearable intracranial haemorrhage.”
- **Inclusion:** Original studies, prospective or retrospective, reporting AI/ML analysis of smartwatch or similar wrist-worn sensors for the detection of SAH, ischemic stroke, or ICH. Must report performance metrics (e.g., sensitivity, specificity, area under the receiver operating characteristic curve [AUC]).
- **Exclusion:** Reviews, editorials, abstracts, unpublished pilots, or conference posters without full text. Animal studies or nonsmartwatch wearables (e.g., helmet sensors) were excluded.

### 2.2. Study selection

Titles and abstracts were screened by 2 reviewers independently. Full-text articles were assessed for eligibility. Discrepancies were resolved by discussion or third-party adjudication. Studies meeting all inclusion criteria were included in data extraction.

### 2.3. Data extraction

From each eligible study, we extracted authors, publication year, study design, setting, sample size, device(s) and sensors used, target condition (ischemic stroke, SAH, ICH), AI/ML model type, features/input data, and performance metrics (sensitivity, specificity, AUC, accuracy, detection time, false-alarm rate, etc). We also noted whether models were validated in separate cohorts.

### 2.4. Risk of bias assessment

We used the Quality Assessment of Diagnostic Accuracy Studies-2 tool for diagnostic accuracy studies. Domains assessed were patient selection (spectrum, sampling), index test (AI model) assessment, reference standard, and flow/timing. Two reviewers scored studies independently, with consensus on conflicts. Due to heterogeneous outcomes (e.g., time to detection vs classification accuracy), no meta-analysis was done.

### 2.5. Data synthesis

Results are summarized narratively in a table. We highlight AI models and key performance outcomes. Limitations and future directions are then discussed.

### 2.6. Ethical review and informed consent

Ethical approval from an institutional review board or ethics committee was not required for this systematic review because it synthesizes findings from previously published studies and does not involve the collection or analysis of identifiable individual patient data from any institution. Accordingly, informed consent was not applicable.

## 3. Results

### 3.1. Study selection

The initial search yielded 872 records after duplicates were removed. Screening titles/abstracts excluded 840 records (mostly unrelated to our focus, e.g., AF detection). We reviewed 32 full-text articles. Of these, 3 studies met the inclusion criteria (all on ischemic stroke detection). No eligible studies were found for AI-smartwatch detection of SAH or other ICHs. The PRISMA flow diagram is shown in Figure 1. The key reasons for exclusion were no AI component, condition not relevant (e.g., only risk prediction), or nonwearable device.

### 3.2. Study characteristics

The 3 included studies focused on acute ischemic stroke and used wearable accelerometers (research-grade wristbands) to detect unilateral arm weakness. No study specifically targeted SAH or ICH. Table 1 summarizes the stroke studies. Two were prospective diagnostic accuracy studies; the third was a feasibility trial. Sample sizes were small (n ≈ 30–180). All studies were in hospital or rehabilitation settings; none were population-based. No study evaluated chronic or recurrent stroke. No study included AF detection as a proxy.

- **Wasselius et al** (2021, Sweden) monitored bilateral wrist accelerometers continuously in 185 subjects (84 acute stroke, 101 controls) for 1 to 7 days postevent. Using convolutional neural networks (CNN; a fully convolutional network, inception time) and classical ML (support vector machine, random forest), they identified unilateral arm paresis with high accuracy.
- **Messé et al** (2023, USA) conducted a case–control study in hospitalized patients (n = 110; 33 strokes with hemiparesis, 167 controls). Bilateral arm accelerometers were used to derive a “stroke classifier algorithm.” Performance was expressed as time-to-detection versus false alarm rate.
- **Esbjörnsson et al** (2022, Sweden) conducted the STROKE ALARM prospective randomized/feasibility study designation (as used in STROKE ALARM PRO1) feasibility trial (n = 30 patients with stroke or transient ischemic attack) using wrist accelerometers and a smartphone app to trigger stroke tests. This study lacked ML models per se; it served primarily to assess user adherence and alarm rates.
- **Jeon et al** (2023, IEEE Access): In this study, two 9-axis inertial measurement unit wristbands recorded overnight motion in 46 healthy subjects and 43 stroke patients with known hemiparesis. A novel deep ensemble (“ensemble deep learning in-sleep stroke detection model”) combined multiple CNNs processing 30- to 180-minute sliding windows to detect “in-sleep” stroke onset. On simulation tests (concatenating normal and stroke data), the best model (ensemble deep learning in-sleep stroke detection model-ResNet50, 120-minute window), achieved a harmonic mean of precision and recall of 0.955 (95% CI 0.950–0.960), accuracy of 93.9% (95% CI 93.3–94.6), sensitivity of 96.8% (95% CI 96.0–97.5), and specificity of 88.5% (95% CI 87.1–89.8). By comparison, a single AlexNet CNN had a sensitivity of 90.7% and a specificity of 78.7%. Thus, multistream deep learning on contralateral wrist motion reliably identified asymmetric movement indicative of stroke, even during sleep.

No studies of SAH or ICH detection by smartwatch were identified. While wearables have been studied for blood pressure (BP) surrogates or trauma hemorrhage monitoring, we found no published algorithm attempting to detect ICH events via wrist sensors.

## 4. AI models and performance

### 4.1. Ischemic stroke

The 2 diagnostic studies employed ML on accelerometer data. Wasselius et al applied deep learning (fully convolutional networks and inception time) and achieved AUCs of 0.947 to 0.994 for detecting stroke-related hemiparesis, depending on observation window length. For example, on 15-minute data windows, AUC ≈ 0.95, rising to ≈ 0.99 on 120-minute windows. Classical methods (support vector machine, random forest) had slightly lower accuracy. Messe et al defined a binary classification (stroke vs nonstroke) by monitoring arm motion. They reported that at a median false alarm rate of 3.6 per day (controls), the median stroke detection time was ~15.0 minutes, whereas a lower alarm rate (1.1/day) yielded ~29.0 minutes of detection. No sensitivity/specificity numbers were reported, but roughly half of stroke cases were detected within ~15 to 30 minutes. The feasibility trial (Esbjörnsson et al) did not yield accuracy metrics due to no actual strokes during monitoring but noted that almost all participants received false alarms (sensor-triggered alerts without stroke), underscoring the challenge of specificity.

Performance metrics are summarized in Table 1. All stroke detection algorithms showed promise in controlled settings. However, these models have not been tested in broader community samples. The high accuracy reported (AUC = 0.99) may be optimistic due to the study design (case–control sampling with clear hemiparesis).

### 4.2. ICH and SAH

No study was identified applying AI-smartwatch methods to detect ICH or SAH. In clinical practice, SAH and ICH require imaging (computed tomography/magnetic resonance imaging) for diagnosis; no noninvasive wearable test currently exists. Conceptually, a severe bleed might cause changes in vitals (e.g., acute hypertension) measurable by a watch, but no validated algorithms are reported.

### 4.3. Risk of bias assessment

All included studies had limitations. Using Quality Assessment of Diagnostic Accuracy Studies-2, the ischemic stroke studies rated high or unclear risk in several domains. Patient selection: The studies used convenience samples of known stroke patients versus controls, raising spectrum bias. Most cases had pronounced hemiparesis, not mild or atypical stroke, limiting generalizability. Index test: The AI algorithms were applied in a research setting, often “on-label” with known outcomes. Blinding of the index test to outcome was adequate. However, thresholds for alarms (as in Messe et al) were chosen ad hoc. Reference standard: Stroke diagnosis was confirmed clinically (and by imaging) for patients, and the absence of stroke was assumed for controls; this gold standard was appropriate. Flow and timing: Study protocols often lacked follow-up; Esbjörnsson’s study had no stroke events to validate. None of the studies externally validated models on independent cohorts. Overall risk of bias was moderate to high due to small size, lack of prospective validation, and selective inclusion of clear-cut cases.

## 5. Discussion

This systematic review found a paucity of published evidence on AI-smartwatch systems for the early detection of cerebrovascular events. Only a few small studies (all on ischemic stroke) exist, and none address hemorrhagic stroke. The stroke studies indicate that wearable motion sensors plus AI can detect acute limb weakness with high accuracy in experimental settings. For example, Wasselius et al reported nearly 0.99 AUC for identifying unilateral arm paresis with a deep learning model. However, these results likely overestimate real-world performance: study subjects had overt deficits and algorithms were tested on data with known labels. In practice, stroke symptoms can be subtle or atypical, and wearable data are noisy (movement artifacts, nonstroke motions).^[10–13]^

The high false-alarm rates observed (e.g., STROKE ALARM trial) highlight a challenge for real-time detection. Users experienced frequent unexplained alerts, which would reduce trust in a commercial system. Balancing sensitivity (early detection) against false positives is difficult. In the study of Messé, allowing ~3.6 false alarms per day yielded a median detection of 15 minutes poststroke, whereas tolerating fewer alarms delayed detection to ~29 minutes. Acceptable trade-offs will depend on risk tolerance. The STROKE ALARM feasibility study even aborted formal evaluation due to excessive false alerts. Future systems must improve specificity, perhaps by fusing multiple signals (see below).

Detecting hemorrhagic strokes via a smartwatch is inherently challenging. SAH/ICH onset often causes sudden headache, vomiting, loss of consciousness – signs not directly measurable by wrist sensors. Some physiological changes occur (e.g., BP spike, heart rate [HR] variability, agitation), but these are nonspecific and may require complex models to interpret.^[8–13]^ Current smartwatches lack brain-specific sensors. As Radiopaedia notes, “CT is almost always the first imaging modality” for ICH. Without direct neural signals, any AI model would rely on indirect proxies (BP via PPG, gait disturbances, etc), which have not been validated. Thus, the lack of studies likely reflects both technical difficulty and the fact that SAH/ICH typically presents to medical attention quickly due to severe symptoms.

### 5.1. Limitations of the evidence

The included studies have multiple limitations: small sample sizes (≤100) and single-center recruitment limit generalizability. Most algorithms were developed and tested on the same or similar data, risking overfitting. None of the stroke models has been prospectively deployed in general populations. Risk of bias is high: patient selection was case–control rather than consecutive sampling and blinding was limited. Performance metrics are heterogeneous (AUC vs detection time), precluding meta-analysis. Importantly, these models were evaluated on acute or inpatient cohorts; none addressed ambulatory monitoring in at-risk individuals or real-time emergency alerts in the community.

### 5.2. Future directions and conceptual model

**Figure 2.**
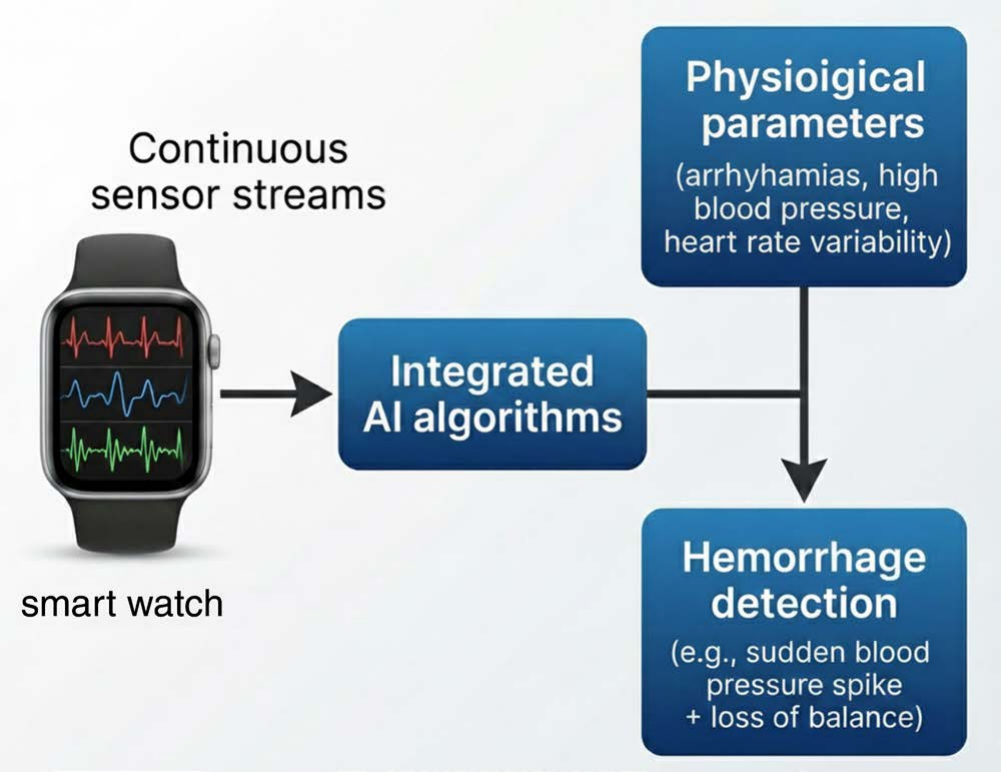
Conceptual model for a multisensor AI-smartwatch system for cerebrovascular event detection. Continuous sensor streams (PPG, ECG, accelerometer, etc) are processed by integrated AI algorithms to identify signatures of stroke (e.g., irregular rhythm + asymmetric motion), or hemorrhage (e.g., sudden blood pressure spike + loss of balance). AI = artificial intelligence, ECG = electrocardiogram, PPG = photoplethysmography.

To move toward practical AI-smartwatch stroke detection, several advances are needed. First, multisensor fusion: a smartwatch should integrate its full sensor suite. For example, PPG/ECG can monitor AF (a stroke risk factor) and sudden HR changes; accelerometers/gyroscopes detect gait disturbance, falls, or limb weakness; microphones or voice analysis might detect speech slurring; and pressure/PPG waveform analysis could infer BP surges. We propose a conceptual model (Fig. 2): raw data streams (HR, ECG, motion, peripheral oxygen saturation) feed into an AI pipeline with layered analytics. Low-level detection of anomalies (e.g., asymmetry in arm swing) triggers higher-level assessment (pattern matching to stroke signatures). A smartwatch might prompt user questions or administer simplified neurological tests via touchscreen when thresholds are crossed. Importantly, edge computing constraints (battery, privacy) require lightweight models or on-device inference with periodic cloud support.

Research must also address user factors. Usability is critical: the wearable must be comfortable for continuous wear and acceptable to lay users. The false-alarm burden seen in trials means algorithms may need adaptive thresholds or multistep confirmation (e.g., combine wrist data with optional smartphone camera tests). Additionally, ethical and regulatory considerations arise: stroke detection algorithms would be classified as medical devices, requiring validation in clinical trials. Data privacy is paramount when monitoring sensitive health data.

Key challenges include signal quality (motion artifacts disrupt PPG/ECG), heterogeneity of stroke presentations, and labeling ground truth (the reference standard is clinical/imaging diagnosis, which may lag wearable signals). AI models risk “black box” bias; transparency and continuous postmarket surveillance are needed. Hardware limitations (e.g., battery life) also constrain continuous high-fidelity monitoring.

### 5.3. Comparison with other technologies

While this review found scant evidence for smartwatch-based stroke detection, related technologies are emerging. For example, wearable neckbands with Doppler ultrasound have been explored for continuous blood flow monitoring, and cuffless PPG wristbands are being studied for BP monitoring. These could eventually complement smartwatch signals. Smartphones have been used to assist stroke recognition via apps and voice/video capture. The synergy of smartphone + wearable might enhance accuracy (e.g., smartphone camera for face asymmetry).

### 5.4. Limitations of this review

Our findings are constrained by the available literature. We are limited to published, peer-reviewed studies; some relevant pilot work might exist unpublished. We also included only English-language sources. Because of heterogeneity and few studies, we did not perform a quantitative synthesis.

## 6. Conclusion

In conclusion, AI-enabled smartwatch platforms demonstrate strong potential for real-time detection of acute intracranial pathologies, particularly stroke, by continuously identifying clinically meaningful asymmetries in limb motion and related physiological perturbations. Extending these capabilities through multimodal sensor fusion, including motion metrics, cardiac rhythm, and BP surrogates derived from PPG/ECG, provides a credible pathway toward recognizing hemorrhagic events such as intracerebral hemorrhage and SAH at symptom onset. When implemented with rigorous prospective validation and specificity-preserving alert logic, such systems can shorten time to triage and definitive imaging, improve access to time-critical therapies, and enhance cost-effectiveness by reducing delays that drive long-term neurological disability.

## Author contributions

**Conceptualization:** Muhammad Mohsin Khan.

**Data curation:** Muhammad Mohsin Khan, Noman Shah.

**Formal analysis:** Muhammad Mohsin Khan, Noman Shah.

**Resources:** Javed Iqbal.

**Software:** Javed Iqbal.

**Investigation:** Brijesh Sathian.

**Methodology:** Brijesh Sathian.

**Supervision:** Simbarashe Samakande, Bipin Chaurasia.

**Validation:** Bipin Chaurasia.

**Visualization:** Bipin Chaurasia.

**Writing – original draft:** Muhammad Mohsin Khan.

**Writing – review & editing:** Bipin Chaurasia.
